# A nomogram for predicting oral frailty in older adults: a small-sample cross-sectional study in Anhui Province, China

**DOI:** 10.3389/fpubh.2026.1698294

**Published:** 2026-03-26

**Authors:** Huan Liu, Ming Zhang, Guangliang Mei, Zhiqing Zhou, Wenyi Jiang, Xiubin Tao, Jun-kai Dou, Li Li

**Affiliations:** 1 Department of Hemodialysis, The First Affiliated Yijishan Hospital of Wannan Medical College (Yijishan Hospital of Wannan Medical College), Wuhu, Anhui, China; 2 School of Innovation and Entrepreneurship, Wannan Medical College, Wuhu, Anhui, China; 3 Key Laboratory of Philosophy and Social Science of Anhui Province on Adolescent Mental Health and Crisis Intelligence Intervention, Hefei Normal University, Hefei, China; 4 The Department of Party Affairs, The First Affiliated Hospital of Wannan Medical College (Yijishan Hospital of Wannan Medical College), Wuhu, Anhui, China; 5 Department of Nursing, The First Affiliated Yijishan Hospital of Wannan Medical College (Yijishan Hospital of Wannan Medical College), Wuhu, Anhui, China; 6 Department of Nursing, Anhui College of Traditional Chinese Medicine, Wuhu, Anhui, China; 7 Nursing Department, Lu’an Hospital of Anhui Medical University, Luan, Anhui, China; 8 Science and Education Department, Lu’an Hospital of Anhui Medical University, Luan, Anhui, China

**Keywords:** depressive symptoms, malnutrition, older adults, oral frailty, subjective cognitive decline

## Abstract

**Background:**

With the population aging, oral frailty among older adults has become an increasingly prominent concern. Oral frailty is a condition that is highly prevalent among older adults and has a significant negative impact on their quality of life. The condition can exacerbate physical frailty among older adults, increasing the risk of disability or death. This study investigated the current status and influencing factors of oral frailty in older adults and identified the potential risk factors for oral frailty.

**Methods:**

The oral frailty of older adults was measured using the Oral Frailty Index-8 (OFI-8) scale. At the same time, their nutritional status was assessed using the Mini Nutritional Assessment–Short-Form (MNA-SF), depressive status was evaluated using the Geriatric Depression Scale (GDS-5), eHealth literacy was measured using the eHealth Literacy Scale, and cognitive status was determined using the Subjective Cognitive Decline Questionnaire (SCD-Q9) scale. First, variables related to oral frailty were preliminarily screened using univariate analyses (the chi-square test and *t*-test). Subsequently, variables with a *p*-value of < 0.05 in the univariate analysis were incorporated into a multivariate binary logistic regression analysis. The forward stepwise selection method (likelihood ratio test) was used to determine the final predictive model to control for overfitting and ensure the model’s parsimony. Based on the final multivariate logistic regression model, an individualized prediction nomogram was constructed. This nomogram converts the regression coefficients of each predictor variable into a 0–100 point scoring system, allowing for intuitive visualization of oral frailty risk by mapping the total score to the predicted probability.

**Results:**

The prevalence of oral frailty among older adults was 46.8% (1,433/3,061). Hospitalization within the past year (*p* = 0.001), depressive symptoms (*p* < 0.001), social isolation (*p* < 0.001), malnutrition (*p* < 0.001), and subjective cognitive decline (*p* < 0.01) were highly correlated with oral frailty in older adults. eHealth literacy (*p* < 0.001) was a protective factor against oral frailty. The area under the curve (AUC) value of the constructed oral frailty prediction model was 0.747 (95% CI: 0.729–0.764), with the calibration curve slope approximating 1. The calibration curve closely aligned with the ideal standard curve, and the quantitative analysis of the H–L value indicated a good fit of the nomogram model (*χ*^2^ = 7.965, *p* = 0.437). This indicates that the final oral frailty prediction model for older adults in Anhui Province demonstrates good predictive performance and can accurately assess the risk of oral frailty in older adults.

**Conclusion:**

This study showed a high prevalence of oral frailty among older adults in China. Hospitalization within the past year, depressive symptoms, malnutrition, and subjective cognitive decline were found to be highly correlated with oral frailty in older adults. Additionally, eHealth literacy was identified as a protective factor against oral frailty in older adults. The government and medical institutions need to develop and implement oral health prevention and management strategies for older adults in China to help reduce the risk of oral frailty.

## Introduction

On a global scale, the number of older adults is increasing at an unprecedented rate, a demographic shift that presents significant public health challenges worldwide. China exemplifies this trend. According to the latest data from the Seventh National Population Census released by the National Bureau of Statistics in May 2021, individuals aged ≥60 years account for 18.70% of the population, while those aged ≥65 years comprising 13.50% (1). Among the multifaceted health issues associated with this aging population, oral frailty has emerged as a critical and growing concern in geriatric health. The World Health Organization has underscored the importance of oral health by recommending its integration into the Universal Health Coverage agenda (2). Oral frailty is defined as the progressive decline in the health and function of the oral system with age, encompassing factors such as tooth loss, oral hygiene status, and overall oral function (3). This decline is significantly associated with adverse health outcomes in older adults. For instance, a study by Kamide et al. demonstrated that oral frailty increases the risk of falls (4), highlighting its far-reaching implications beyond oral health itself.

In addition, Puranen et al. (5) indicated that oral frailty was associated with decreased health-related quality of life (HRQoL) and mortality. Consequently, oral frailty not only has a significant impact on the health status of older adults but also imposes a heavy burden on society and the healthcare system. Oral frailty is similar to frailty (6); the oral frailty process can be stopped or even reversed to a certain extent if appropriate health interventions and preventive measures are implemented early. Therefore, early screening and development of targeted interventions for oral frailty are important for reducing future adverse health outcomes. It is necessary to prevent oral frailty among older adults and the associated factors that could be intervened on. One such factor is depressive symptoms. Depressive symptoms refer to the long-term occurrence of depressive emotions and the loss of pleasure or interest in activities (7).

Depressive symptoms are a common mental health problem among older adults and can negatively affect their physical health, including the deterioration of oral health. Research has shown that depression can indirectly affect oral health through neglecting personal care and hygiene, which may lead to an increase in the incidence of dental caries and periodontal disease and ultimately lead to tooth loss and impaired oral function (8). Hu et al. (9) have reported that the risk of oral frailty increased by 4.89 times in older adults with depressive symptoms. Previous studies have pointed out that depressive symptoms are closely associated with oral frailty symptoms, such as swallowing disorders and tooth loss (10–12).

Malnutrition, defined as an imbalance of energy, protein, and other nutrients, can have serious implications for older adults, affecting their physical and cognitive functioning and leading to increased vulnerability to diseases and higher mortality rates (13). It is important to note that malnutrition in older adults is often associated with multiple comorbidities, including frailty, which is characterized by diminished physiological reserves and an increased risk of adverse health outcomes (14). Frailty, a significant concern in geriatric health, is also associated with a decline in oral function, manifesting as difficulty in chewing, swallowing, and maintaining oral hygiene. Subjective cognitive decline (SCD) refers to the subjective experience of the decline in cognitive ability, indicating that there is no objective impairment in cognitive assessment (15). It often manifests as an early indicator of cognitive impairment and has been associated with factors such as age, education, and overall mental health. Older adults with cognitive impairment often neglect or forget oral hygiene and may face obstacles to regularly seeking high-quality dental care (16), resulting in a higher oral bacterial load and increased levels of inflammation. On the one hand, a higher oral bacterial load increases the risk of tooth decay and loss (17) and, consequently, the risk of oral frailty.

With the significant acceleration of population aging, the social isolation of older adults has been increasingly recognized as a prominent public health concern (18). Social isolation is typically defined as having infrequent social contact or lacking social integration (19). Previous research has shown that social isolation in older adults predicts a range of adverse health outcomes (20, 21). Due to social isolation, a majority of older adults have reduced contact with the outside world and delayed medical treatment, which poses additional challenges for their health management. The lack of regular oral examinations and treatments not only increases the incidence of oral diseases but may also aggravate existing oral health problems.

The concept of eHealth literacy has developed from traditional health literacy and is defined as an individual’s ability to seek, find, understand, and evaluate health information from electronic sources and to apply the acquired knowledge to solve or address health problems (22). In response to the challenges posed by an aging population, there is a growing interest in eHealth literacy as a proactive approach to improving the health management of the general resident population (23). There is no doubt that in today’s online environment, eHealth literacy for older adults plays an increasingly important role in overall health management and health promotion. Therefore, the relationship between eHealth literacy and oral frailty in older adults remains a key research topic that is actively being explored.

Based on existing literature, we hypothesized that depressive symptoms, malnutrition, subjective cognitive decline, and social isolation are associated with oral frailty in older adults. We also postulated that having been hospitalized in the past year might be a risk factor, while digital health literacy could serve as a protective factor.

To move beyond merely identifying associated factors and to provide a quantifiable clinical tool, this study aimed to integrate these potential predictors to develop and validate a nomogram model. This model enables the individualized prediction of oral frailty risk in older adults. By visually presenting the weight of each contributing factor, the nomogram is intended to assist healthcare professionals in early identification, stratifying high-risk individuals and formulating targeted intervention strategies. Ultimately, the application of this model could contribute to reducing the prevalence of oral frailty and improving the overall health and wellbeing of the older population.

## Methods

### Samples and procedure

This was a cross-sectional study with provincial-level representation conducted in Anhui Province, eastern China. The study was designed and reported in full accordance with the Strengthening the Reporting of Observational Studies in Epidemiology (STROBE) guidelines. Participants were recruited using a convenience sampling strategy between July and August 2024.

Prior to data collection, all investigators completed a standardized training program to ensure consistency in understanding the survey instruments, scoring criteria, communication techniques, and procedural protocols. Following the obtaining of informed consent, participants received a self-administered electronic questionnaire. For individuals with limited literacy or reading difficulties, researchers conducted face-to-face interviews to assist in completing the questionnaire.

We emphasized that participation was entirely voluntary, and participants retained the right to withdraw unconditionally at any point without penalty. Electronic informed consent was obtained from all participants before their involvement in the study. The consent form detailed the study’s objectives, data collection procedures, measures to ensure confidentiality, and the anonymous nature of data handling. Participants were informed that the research aimed to gather information on oral health status, with all responses kept confidential and anonymized.

**Figure 1:**
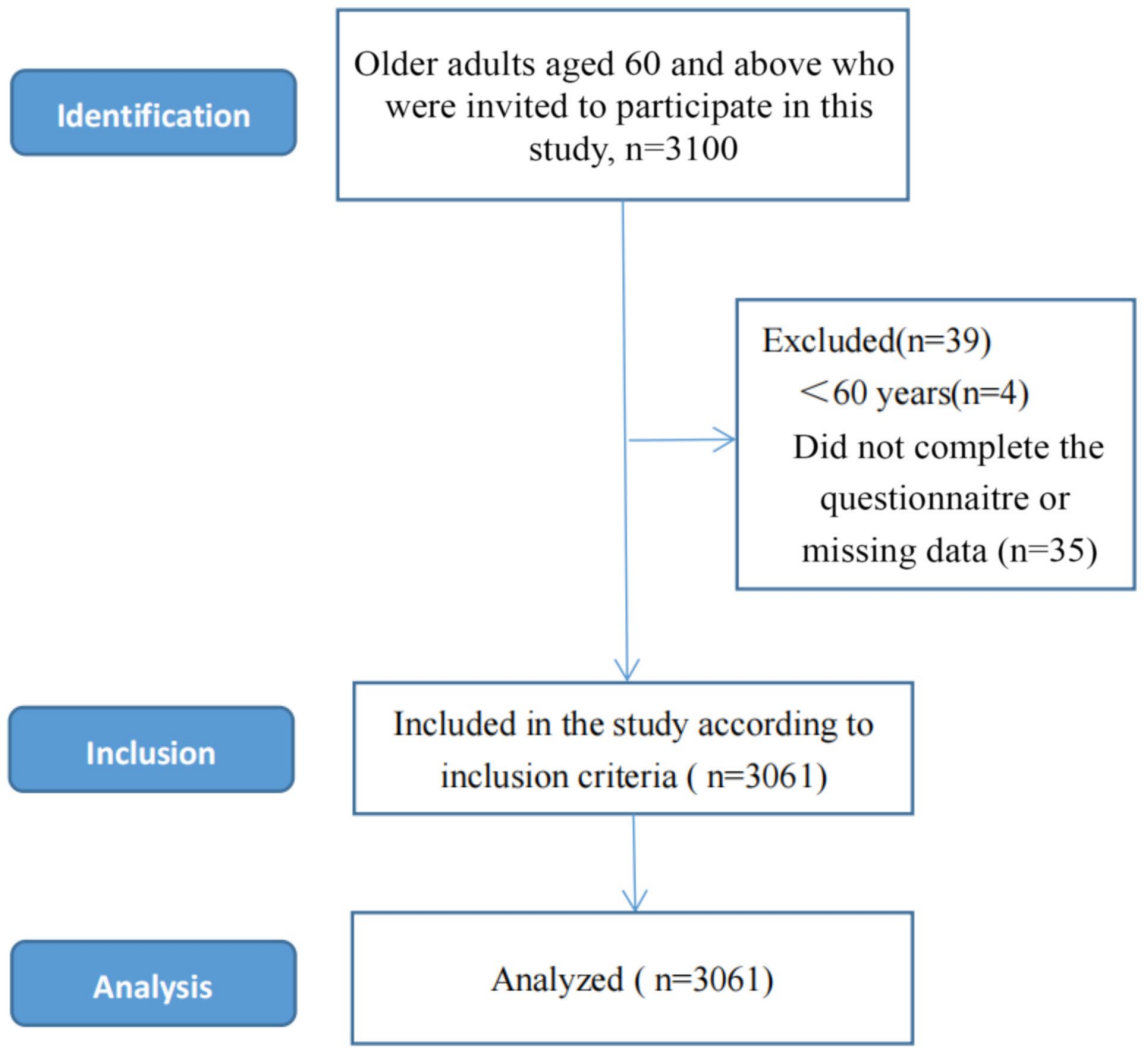
Sample selection process for this cross-sectional study.

A total of 3,100 electronic questionnaires were distributed, with 3,061 valid responses returned, yielding a validity rate of 98.7%. The inclusion criteria were as follows: (i) provision of informed consent and voluntary participation; (ii) clear consciousness and no verbal communication barriers; (iii) no diagnosed neurological or psychiatric disorders; and (iv) age 60 years or older (see Figure 1 for the participant flow).

### Population context and representativeness

To contextualize the sample within the target population, we provide the following demographic details. According to the Seventh National Population Census of China, Anhui Province has a permanent resident population of approximately 61.27 million, of which approximately 11.72 million (19.1%) are aged 60 years and older. The final analytical sample of 3,061 older adults in this study therefore represents approximately 0.026% of the estimated total older adult population (aged 60+) in the province. While convenience sampling limits the strict generalizability of the findings, this sample size provides a substantive basis for preliminary model development and correlation analysis within the studied region. The inclusion of a broad range of variables, as noted, facilitated the exploration of significant associations relevant to oral frailty.

## Measurements

### Demographic characteristics

A structured questionnaire was used to collect socially and demographically relevant information from the participants. The questionnaire was developed based on a comprehensive review of the literature on older adults and in consultation with relevant epidemiological experts. The demographic questionnaire obtained information on age, sex, education level, place of residence, employment status, marital status, living arrangements, and lifestyle habits.

Tanaka et al. developed the Oral Frailty Index-8 (OFI-8) (24). There are eight items on this scale; the first three questions are scored from 0 to 2 points each, and the last five questions are scored from 0 to 1 point. The total score ranges from 0 to 11 points. Higher scores indicate a greater risk of oral frailty, with a score of ≥4 considered indicative of oral frailty. In China, the OFI-8 scale is widely used as a screening tool to assess the degree of oral frailty in older adults in clinical and research settings (25). The Cronbach’s *α* value of the OFI-8 was 0.84 in this study.

The Mini Nutritional Assessment–Short-Form (MNA-SF) is a unidimensional scale consisting of six items: food intake, weight loss, mobility, psychological distress or acute illness, neuropsychological problems, and body mass index (BMI). Based on the total MNA-SF score, an individual’s nutritional status is categorized into three groups: malnourished (0–7 points), at risk of malnutrition (8–11 points), and well-nourished (12–14 points) (26). The MNA-SF has been widely validated among Chinese older adults and demonstrates excellent reliability and validity (27). In this study, MNA-SF scores ≤11 indicated poor nutritional status or risk of malnutrition (28). The Cronbach’s *α* value of the MNA-SF in this study was 0.91.

Social isolation was assessed using three questions: “Are you currently living with others?” (living alone = 1), “Do you visit friends or family at least once a month?” (less than once a month = 1), and “How often do you attend the following [parties/social events] per week?” (less than once a week = 1). We obtained a total score for the 3-item social isolation items by adding the scores for each item. The total social isolation score ranges from 0 to 3, with a higher total score indicating more severe social isolation. In addition, possible social isolation is indicated when the total score is 2 or above (29).

The Geriatric Depression Scale (GDS-5) was selected as the measure of depressive symptoms in older adults. It was developed by Hoyl et al., based on the 15-item Geriatric Depression Scale (GDS-15) (30). Participants could answer “yes” or “no” to each item and were scored 0 or 1, respectively. Higher GDS-5 total scores indicate more severe depressive symptoms. The Chinese version of GDS-5 has demonstrated good reliability and validity for assessing depressive symptoms among Chinese older adults (31). The Cronbach’s *α* value of the GDS-5 was 0.85 in this study.

The Electronic Health Literacy Scale (EHEALS) is a measurement standard for evaluating electronic health literacy, originally developed by Norman and Skinner in 2006 (32). Guo et al. adapted and revised the scale for Chinese populations in 2014 (33). The scale consists of 8 items, which are divided into three areas: testing the network’s ability to implement information and medical services, testing evaluation capabilities, and testing decision-making capabilities. Participants responded using a 5-point Likert scale (ranging from 1 = strongly disagree to 5 = strongly agree), yielding a total score ranging from 8 to 40 points. Higher scores indicate greater eHealth literacy. A score of 32 or lower was defined as poor eHealth proficiency, whereas a score of 32 or higher was defined as satisfactory eHealth proficiency. The Cronbach’s *α* value of the EHEALS was 0.86 in this study.

The Subjective Cognitive Decline Questionnaire (SCD-Q9) is a brief tool designed for screening subjective cognitive decline (SCD). Introduced and translated into Chinese by Hao et al. (34), localization studies have confirmed that the SCD-Q9 is appropriate for the Chinese population (35). The scale encompasses two dimensions: overall memory ability and daily activity ability, consisting of a total of nine items. It uses a scoring system with two or three levels, ranging from “yes” to “no” or from “often” to “never.” Each item is scored from 0 to 1 point, with all items scored positively. The total scale score ranges from 0 to 9 points, with a cutoff of 3 points distinguishing between normal cognitive function and subjective cognitive decline. Higher scores indicate more severe subjective cognitive decline. The Cronbach’s *α* value of the SCD-Q9 was 0.91 in this study.

Descriptive statistics were used to characterize participants. Continuous data were reported as mean and standard deviation (SD), while categorical data were reported as frequencies and percentages. A *χ*^2^ test was used to analyze the association between each participant’s demographic characteristics and oral frailty. Variables with a *p*-value of < 0.05 in the univariate analysis were included as independent variables in the binary logistic regression analysis to identify factors associated with oral frailty. A multivariate logistic regression analysis was then applied to examine the association between oral frailty and its influencing factors, with results expressed as odds ratios (ORs) and 95% confidence intervals (CIs) to establish a risk prediction model.

This study used R software for the development and internal validation of the prediction model. Initially, a multivariate logistic regression model was constructed using the entire study population, and a nomogram was plotted to enable individualized prediction of the risk of oral frailty. The model’s performance was assessed for discriminative ability through the receiver operating characteristic (ROC) curve and the area under the curve (AUC). Calibration curves were plotted, and the Hosmer–Lemeshow goodness-of-fit test was used to evaluate the model’s calibration. A decision curve analysis (DCA) was used to assess the clinical net benefit of the model at different threshold probabilities. The development process of the nomogram model is as follows: ① variables associated with oral frailty were identified through the univariate analysis (*p* < 0.05) and included in the multivariate logistic regression analysis and ② a logistic regression model was constructed using the rms package in R software, and a nomogram was plotted. Each variable’s corresponding score was linearly mapped to a 0–100 point scale, and the total score corresponded to the risk scale at the bottom, enabling direct estimation of the individual probability of oral frailty occurrence. Model validation was conducted using the bootstrap resampling method (1,000 times) for internal validation. The optimism-corrected AUC and calibration slope were calculated to assess the model’s discrimination and calibration. Bootstrap calibration curves were plotted, and the Hosmer–Lemeshow test *p*-value was used to evaluate the agreement between the model’s predicted probabilities and the actual observed risks. Furthermore, a decision curve analysis was used to compare the clinical net benefits of the model at different threshold probabilities, thereby assessing its clinical utility.

## Results

### Description of the sample

Table 1 presents the demographic characteristics of the older adults. Among the 3,061 older adults, 52.4% (*n* = 1,604) were male, and 47.6% (*n* = 1,457) were female. The age of the medical college students participating in this study ranged from 60 to 99 years old, with a mean age of 69.85 ± 7.83. A total of 977 (31.9%), 761 (24.9%), and 1,323 (43.2%) participants resided in the county, town, and city, respectively. Detailed sociodemographic characteristics of participants are presented in Table 1.

**Table 1:** Univariate analysis of the participants’ demographic characteristics (*N* = 3,061).

Variables	Grouping	*n*	(%)
Sex	Male	1,604	52.4
	Female	1,457	47.6
Place of residence	Rural	977	31.9
	Town	761	24.9
	City	1,323	43.2
Areas	North Anhui	1,390	45.4
	Central Anhui	864	28.2
	Southern Anhui	807	26.4
Age group	60–69	1,781	58.2
	70–79	903	29.5
	≥80	377	12.3
Education level	Illiteracy	1,138	37.2
	Elementary school	750	24.5
	Junior high school	445	14.5
	High school	269	8.8
	Technical secondary school	150	4.9
	Junior college	95	3.1
	Bachelor’s degree	127	4.1
	Master’s degree or above	87	2.8
Marital status	Married	2,178	71.2
	Separated	131	4.3
	Divorced	113	3.7
	Widowed	469	15.3
	Single	84	2.7
	Others	86	2.8
Hospitalization within the past year	No	1,738	56.8
	Yes	1,323	43.2
Alcohol consumption	No	1,387	45.3
	Already quit drinking	891	29.1
	Yes	783	25.6
Smoking status	No	1,645	53.7
	Already quit smoking	891	29.1
	Yes	525	17.2

### Univariate analysis of risk factors for oral frailty in older adults

In this study, the prevalence of oral frailty in older adults was 46.8% (1,433 of the 3,061 participants). Significant differences were observed across age groups, history of hospitalization within the past year, presence of chronic diseases, depressive symptoms, malnutrition, social isolation, eHealth literacy, and subjective cognitive decline (*p* < 0.05, Table 2).

**Table 2:** Univariate analysis of factors associated with oral frailty.

Variables	Grouping	Non-oral frailty (*n* = 1,628)	Oral frailty (*n* = 1,433)	*χ^2^*	*P*
Sex	Male	865 (53.9%)	739 (46.1%)	0.746	0.388
	Female	763 (52.4%)	694 (47.6%)		
Place of residence	Rural	545 (55.8%)	432 (44.2%)	5.183	0.075
	Town	383 (50.3%)	378 (49.7%)		
	City	700 (52.9%)	623 (47.1%)		
Areas	North Anhui	737 (53.0%)	653 (47.0%)	0.078	0.962
	Central Anhui	463 (53.6%)	401 (46.4%)		
	Southern Anhui	428 (53.0%)	379 (47.0%)		
Age group	60–69	1,011 (56.8%)	770 (43.2%)	28.418	<0.001
	70–79	456 (50.5%)	447 (49.5%)		
	≥80	161 (42.7%)	216 (57.3%)		
Hospitalization within the past year	No	996 (57.3%)	742 (42.7%)	27.441	<0.001
	Yes	632 (47.8%)	691 (52.2%)		
Chronic disease	No	981 (54.7%)	812 (45.3%)	4.056	0.044
	Yes	647 (51.0%)	621 (49.0%)		
Alcohol consumption	No	741 (53.4%)	646 (46.6%)	0.859	0.651
	Already quit drinking	463 (52.0%)	428 (48.0%)		
	Yes	424 (54.2%)	359 (45.8%)		
Smoking status	No	900 (54.7%)	745 (45.3%)	3.512	0.173
	Already quit smoking	462 (51.9%)	429 (48.1%)		
	Yes	266 (50.7%)	259 (49.3%)		
Education level	Illiteracy	590 (51.8%)	548 (48.2%)	11.384	0.123
	Elementary school	386 (51.5%)	364 (48.5%)		
	Junior high school	261 (58.7%)	184 (41.3%)		
	High school	144 (53.5%)	125 (46.5%)		
	Technical secondary school	84 (56.0%)	66 (44.0%)		
	Junior college	48 (50.5%)	47 (49.5%)		
	Bachelor	75 (59.1%)	52 (40.9%)		
	Master’s degree or above	40 (46.0%)	47 (54.0%)		
Depressive symptoms	No	1,203 (61.9%)	741 (38.1%)	161.853	<0.001
	Yes	425 (38.0%)	692 (62.0%)		
Malnutrition	No	711 (75.8%)	227 (24.2%)	277.786	<0.001
	Yes	917 (43.2%)	1,206 (56.8%)		
Social isolation	No	1,118 (59.2%)	769 (40.8%)	72.621	<0.001
	Yes	510 (43.4%)	664 (56.6%)		
eHealth literacy	No	1,328 (49.5%)	1,354 (50.5%)	117.173	<0.001
	Yes	300 (79.2%)	79 (20.8%)		
Subjective cognitive decline	No	741 (77.9%)	210 (22.1%)	338.947	<0.001
	Yes	887 (42.0%)	1,223 (58.0%)		

### Correlations between measurement variables

**Figure 2:**
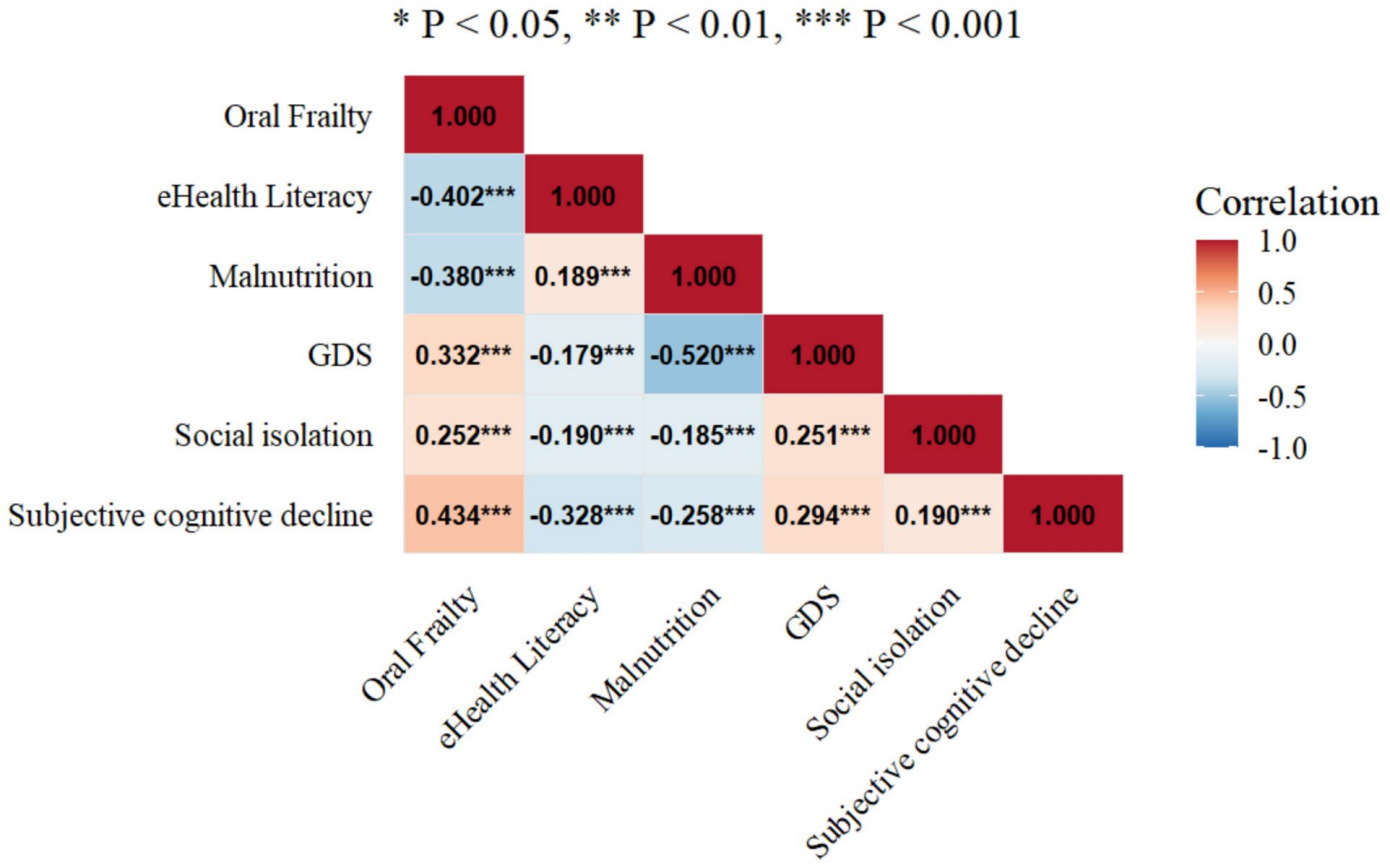
Correlations between oral frailty, eHealth literacy, malnutrition, GDS, social isolation, and subjective cognitive decline.

**Figure 3:**
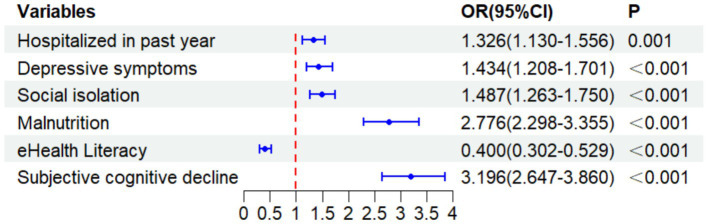
Forest plot: factors affecting oral frailty using a binary logistic regression analysis.

As shown in Figure 2, there was a significant positive correlation between malnutrition, GDS, social isolation, subjective cognitive decline, and oral frailty (*p* < 0.01). In contrast, eHealth literacy was significantly negatively correlated with oral frailty (*p* < 0.01) (Figure 3).

### Binary analysis of influencing factors of oral frailty

A binary logistic regression analysis was performed to explore the influencing factors of oral frailty. Predictors were selected based on the univariate analysis, with those showing a significance level of *p* < 0.05 included in the multivariate model. The independent variables included age group, history of hospitalization within the past year, presence of chronic diseases, depressive symptoms, malnutrition, social isolation, eHealth literacy, and subjective cognitive decline, whereas oral frailty (grouped as 1 = non-oral frailty, 2 = oral frailty) was used as the dependent variable. Before the final regression, multicollinearity among the independent variables was assessed using the variance inflation factor (VIF). All VIF values were below 2.0, indicating no significant multicollinearity that would violate the model assumptions. The results indicated that older adults who had been hospitalized within the past year were more likely to experience oral frailty (OR = 1.326, 95% CI: 1.130–1.556). Older adults with depressive symptoms, social isolation, malnutrition, and subjective cognitive decline were more likely to experience oral frailty (OR = 1.434, 95% CI: 1.208–1.701; OR = 1.487, 95% CI: 1.263–1.750; OR = 2.776, 95% CI: 2.298–3.355; OR = 3.196, 95% CI: 2.647–3.860). In contrast, eHealth literacy was identified as a protective factor for oral frailty (OR = 0.400, 95% CI: 0.302–0.529) among older adults (Table 3).

**Table 3:** Binary logistic regression analysis of factors associated with oral frailty.

Variables	*β*	SE	Wald	*p*-value	OR	95%CI
Hospitalization within the past year	0.282	0.082	12.003	0.001	1.326	1.130–1.556
Depressive symptoms	0.360	0.087	16.990	<0.001	1.434	1.208–1.701
Social isolation	0.397	0.083	22.679	<0.001	1.487	1.263–1.750
Malnutrition	1.021	0.097	111.934	<0.001	2.776	2.298–3.355
eHealth literacy	−0.917	0.143	40.910	<0.001	0.400	0.302–0.529
Subjective cognitive decline	1.162	0.096	145.660	<0.001	3.196	2.647–3.860
Constant	−2.009	0.114	312.933	<0.001	0.134	

Logit(P) = −2.009 + 0.282 × (hospitalized) + 0.360 × (depressive) + 0.397 × (social isolation) + 1.021 × (malnutrition) − 0.917 × (eHealth literacy) + 1.162 × (cognitive decline).

### Construction of predictive models

**Figure 4:**
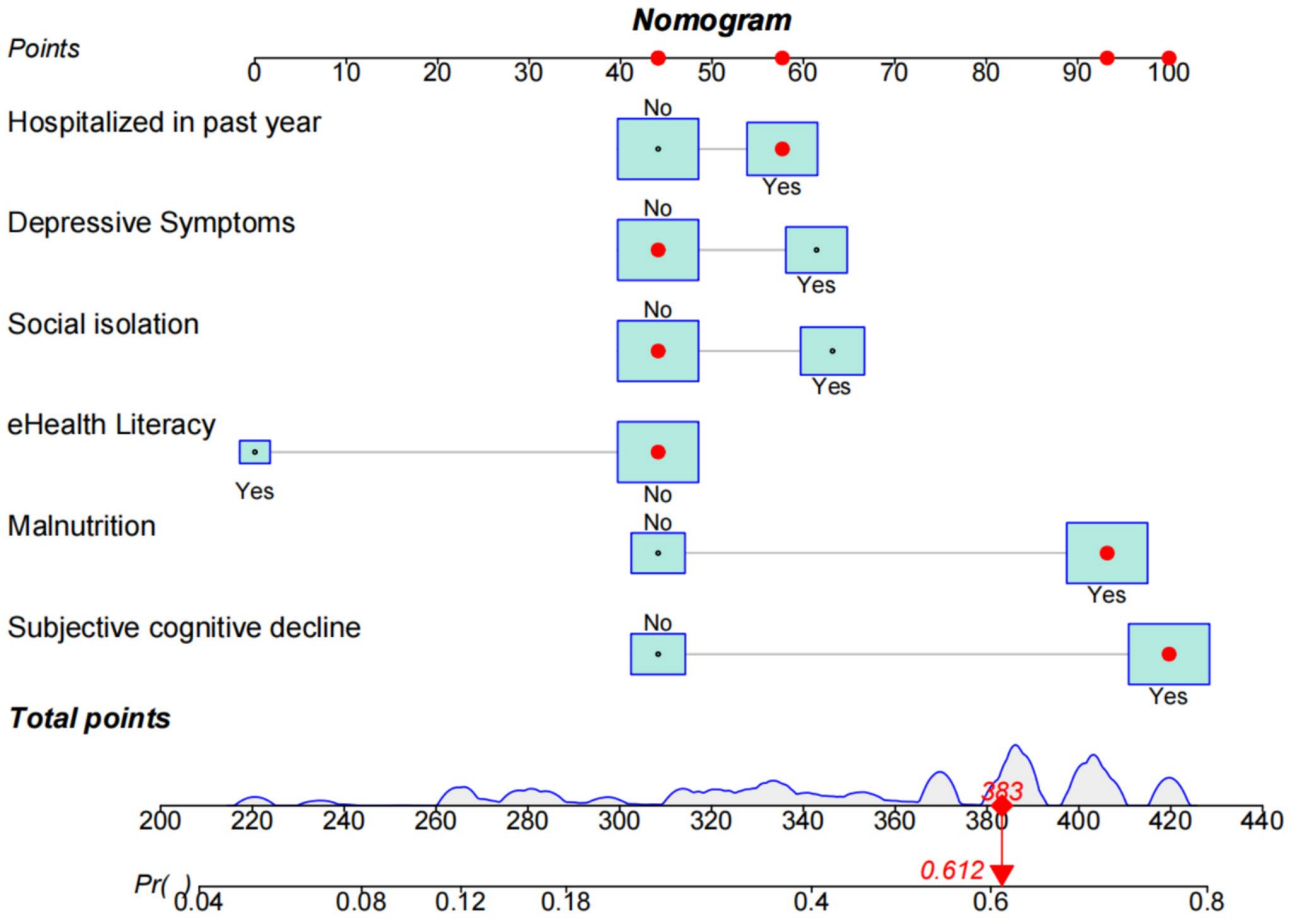
Nomogram prediction model for oral frailty risk in older adults.

According to the binary logistic regression results, factors including hospitalization in the past year, depressive symptoms, social isolation, eHealth literacy, malnutrition, and subjective cognitive decline are independent influencing factors of oral frailty. The rms package was used to construct the model, and the replot package was used for model visualization. To quantify the risk assessment of individual oral frailty, a personalized scoring nomogram was generated to predict the probability of individual oral frailty using six parameters, with an arrow indicating an example. For instance, a subject with subjective cognitive decline, malnutrition, hospitalization within the past year, poor digital health literacy, and neither depression nor social isolation corresponded to a predicted probability of oral frailty of 61.2%, as shown in Figure 4.

### Model assessment

**Figure 5:**
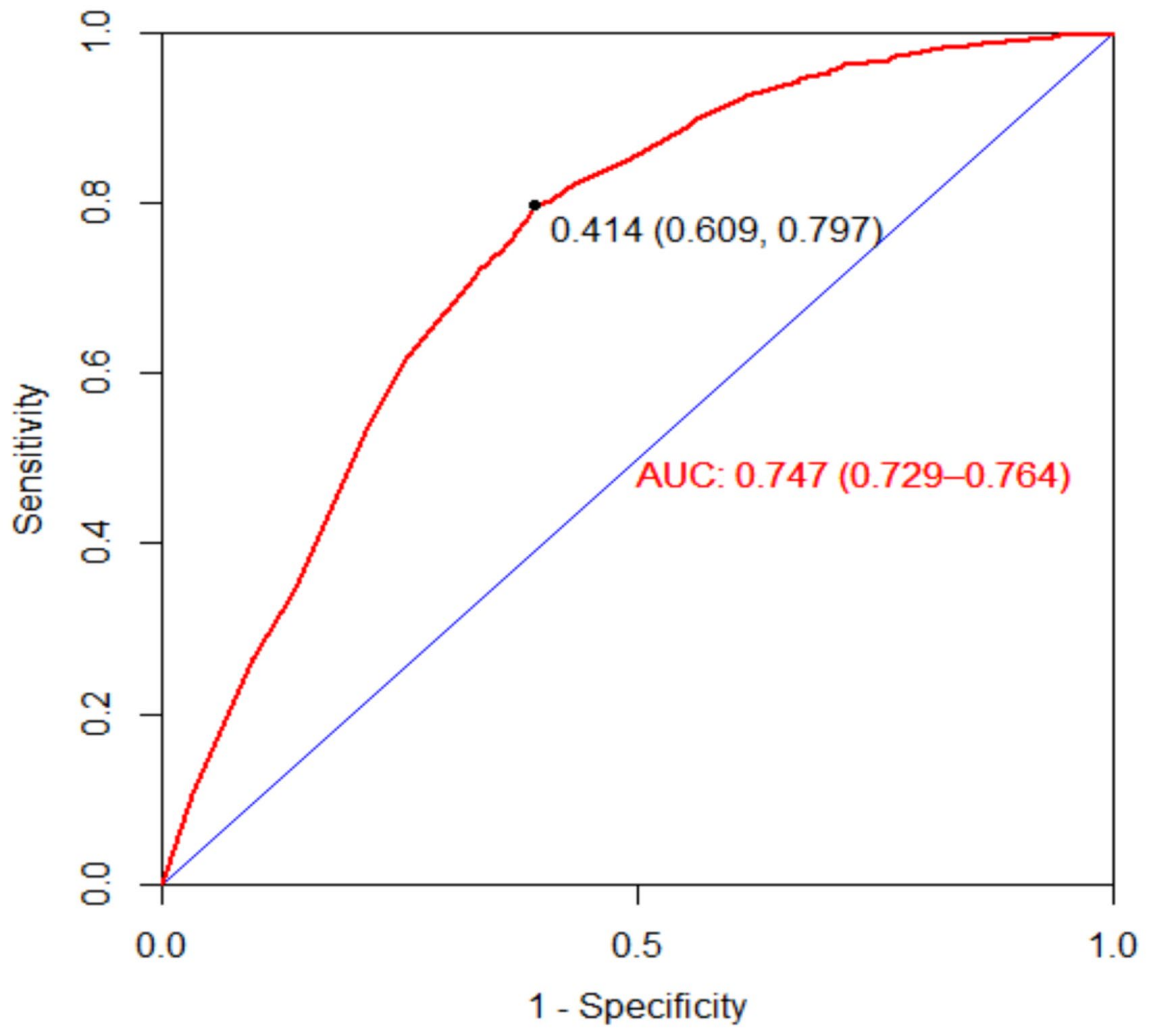
ROC curve of the nomogram prediction model for oral frailty risk.

**Figure 6:**
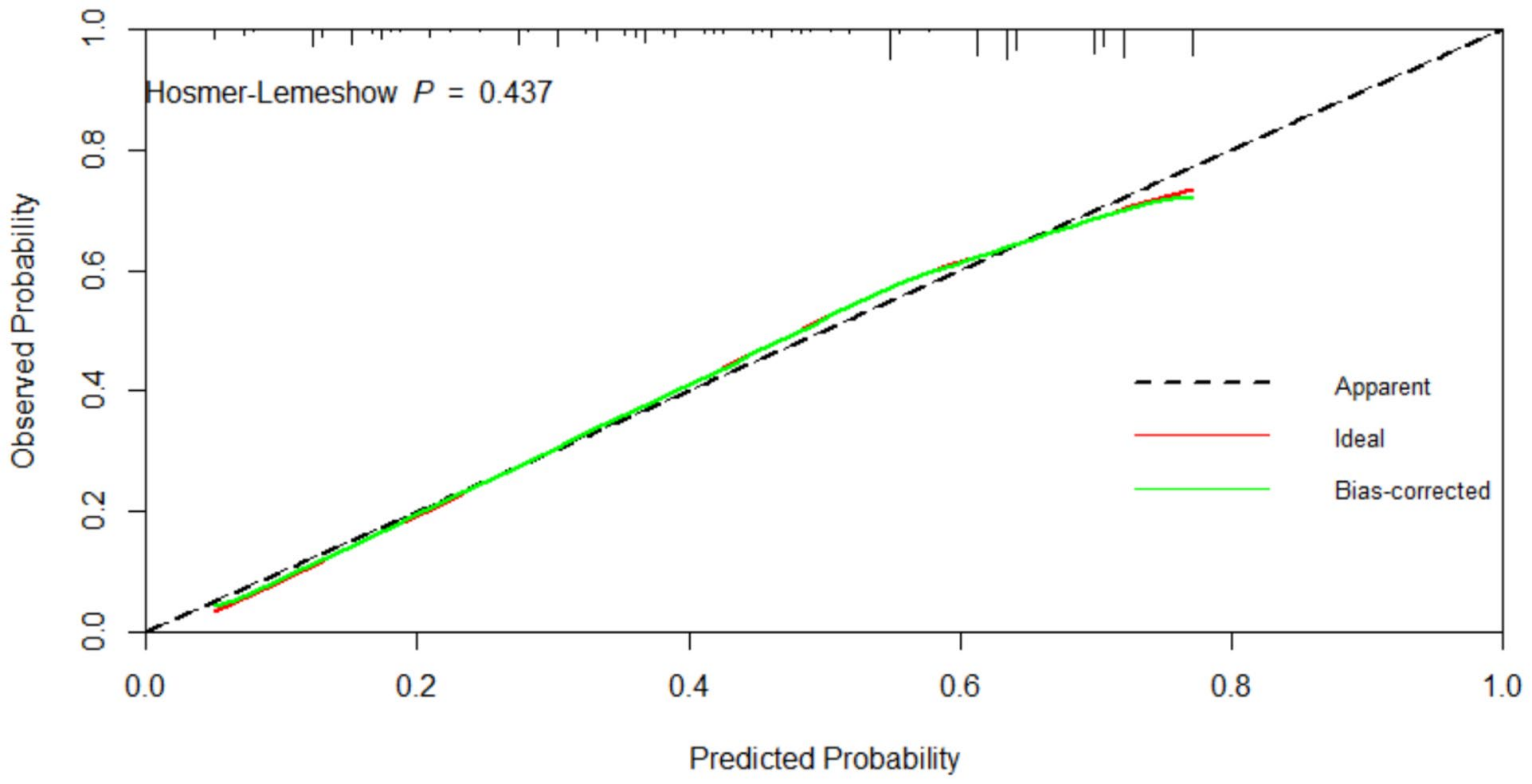
Calibration curve of the nomogram prediction model for oral frailty risk.

**Figure 7:**
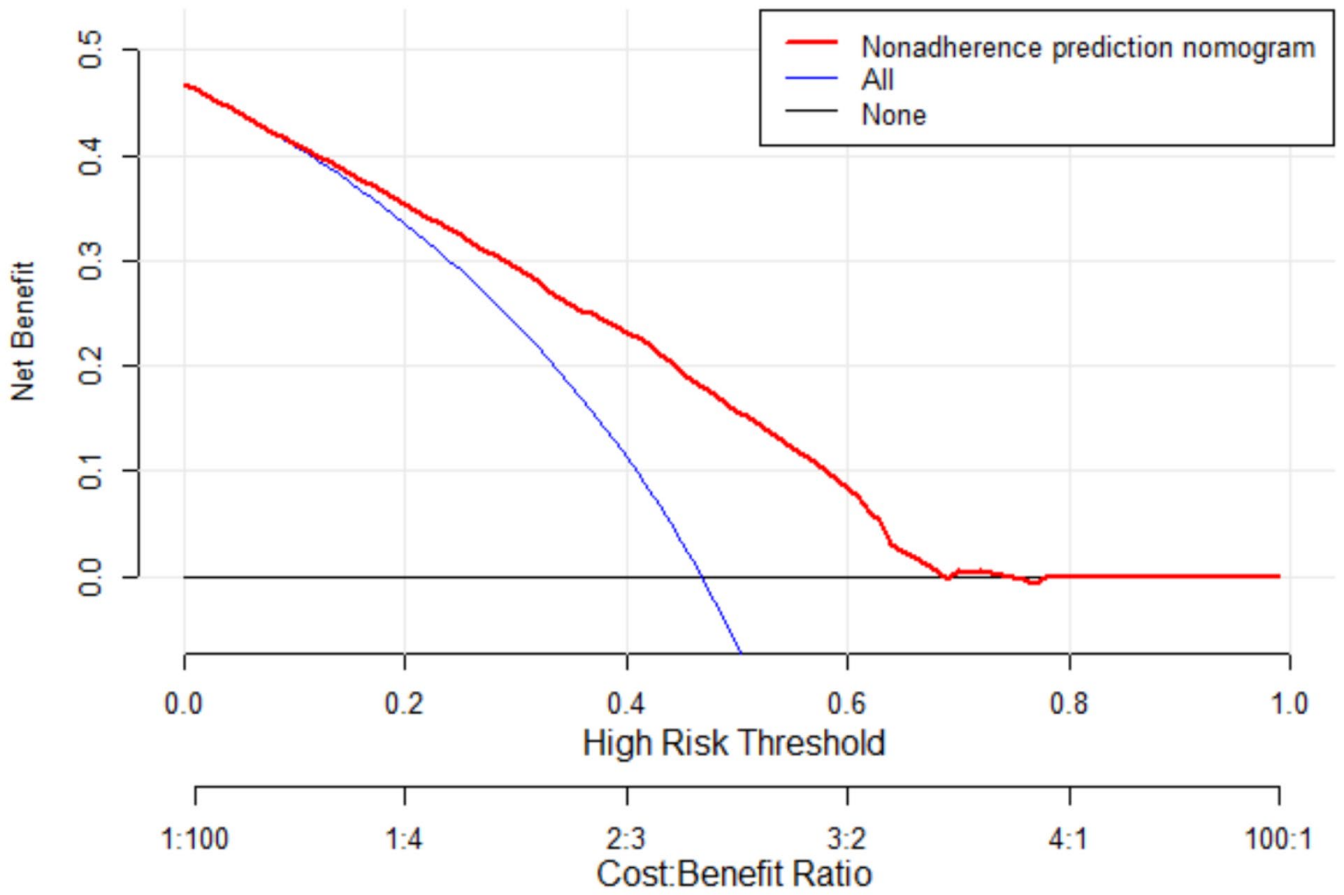
Decision curve of the nomogram prediction model for oral frailty risk.

The bootstrap resampling method was used to validate the constructed model, with 1,000 repetitions for internal validation of the nomogram model. The model’s C-index was 0.747 (95% CI: 0.731–0.764), indicating good discriminative ability of the model. Additionally, the AUC value was determined to evaluate the discrimination of the nomogram. As shown in Figure 5, the AUC value for predicting oral frailty by the nomogram was 0.747 (95% CI: 0.729–0.764), confirming the robust discriminative ability of the model. To determine the optimal cutoff probability for classifying individuals as high- or low-risk, the Youden Index (sensitivity + specificity - 1) was maximized. The optimal cutoff probability was 0.414. At this cutoff, the nomogram demonstrated a sensitivity of 79.9% and a specificity of 60.9% in the validation cohort. The slope of the calibration curve approximated 1, and the curve closely aligned with the ideal reference line. Quantitative analysis of the H–L test indicated a good fit of the nomogram model (*χ*^2^ = 7.965, *p* = 0.437), suggesting a strong consistency between the predicted probability of oral frailty in older adults and the observed occurrence in older adults, as detailed in Figure 6. The clinical utility of the nomogram risk prediction model was evaluated using decision curve analysis (DCA), as shown in Figure 7. According to the decision curve, when the model’s threshold probability is set between 18 and 65%, the decision curve lies above and to the right of the “none” and “all” reference lines, indicating that the nomogram provides a favorable net benefit for patients and demonstrates clinical utility.

## Discussion

This study found that the prevalence of oral frailty among older adults was 46.8%, which is consistent with the findings of a meta-analysis by Li et al. (36). Previous studies have reported that the prevalence of oral frailty among older adults in Chinese communities ranges from 33.8 to 69.0% (37, 38). The meta-analysis by Li et al. (36) reported a prevalence of 45.9% among Chinese residents, which is higher than that reported in developed countries such as Japan and Finland. There are several reasons for this. First, the majority of surveys in China use the OFI-8 tool, which typically yields higher prevalence rates than the Oral Frailty Index-6, resulting in higher measured prevalence. Second, as a developing country, the oral health status of older adults in China may indeed be poorer. As the country with the largest number of older adults in the world, China should pay more attention to the oral health of its older adults and implement early interventions for oral frailty to prevent its potentially negative consequences.

The findings of this study suggest that oral frailty is not an isolated condition, but rather a critical intersection within the complex network of physiological, nutritional, and psychosocial decline that is characteristic of the aging process. Our analysis supports a model in which oral frailty acts as both a consequence and a catalyst within the broader frailty syndrome, with malnutrition serving as a central mediator and psychosocial factors as significant contributors to its onset and progression.

Conceptually, oral frailty may represent a prodromal or core component of physical frailty. The identified factors—recent hospitalization, cognitive decline, depressive symptoms, and social isolation—are all established correlates of general frailty. This overlap indicates that oral dysfunction is not merely a parallel issue but rather is embedded within the same systemic vulnerability. For instance, the heightened risk following hospitalization (OR = 1.326) likely reflects the acute stress, functional decompensation, and routine disruption that can unmask or accelerate latent frailty across multiple domains, including the oral cavity (39, 40). The oral system, with its demands for fine motor coordination and consistent self-care, may be among the first systems to manifest functional decline under such stress.

Central to this framework is the potent bidirectional relationship between oral frailty and malnutrition (OR = 2.776), which appears to form a vicious cycle crucial to understanding overall health deterioration. On the one hand, malnutrition, through pathways such as protein deficiency and micronutrient depletion, directly compromises oral mucosal integrity, salivary function, and immune defenses, thereby precipitating oral frailty. On the other hand, established oral frailty—characterized by chewing difficulty, swallowing problems, and pain—restricts food selection and intake, thereby exacerbating nutritional deficits (41). This cycle creates a conduit through which oral health directly impacts systemic resilience, sarcopenia, and immune function, thereby fueling the progression of broader physical frailty (42, 43).

The strong associations between cognitive decline (OR = 3.196) and depressive symptoms (OR = 1.434) further illuminate the psychosocial dimensions of this framework. These relationships are likely multidirectional. Cognitive impairment can lead to neglect of oral hygiene and barriers to accessing care, directly causing oral health decline (16). Conversely, the chronic inflammation and nutritional deficits associated with poor oral health may contribute to neuroinflammation and cognitive decline. Similarly, depressive symptoms can sap the motivation necessary for consistent oral self-care (44), while the pain, social embarrassment, and functional limitations of oral frailty can themselves precipitate or worsen depressive states (45). Social isolation (OR = 1.487) exacerbates this cluster by reducing social cues for meal regularity and oral care, while also limiting the cognitive stimulation that helps maintain oral motor function through communication (46).

An independent association was observed between higher eHealth literacy and a lower risk of oral frailty (OR = 0.400). This exploratory finding suggests that, after adjusting for included covariates such as education, the perceived capacity to access and apply digital health information may be associated with better oral health outcomes. It is plausible that this capacity could help some older adults to bridge health knowledge gaps, navigate preventive care options, and sustain self-care practices, potentially buffering against certain risk factors such as social isolation (47). However, this association may be subject to residual confounding from unmeasured socioeconomic factors and requires further investigation.

### Limitations

This study has several limitations that should be acknowledged. First, the cross-sectional design precludes the establishment of temporal or causal relationships among the identified factors and oral frailty. Future studies employing probabilistic sampling methods (e.g., stratified, random sampling) across diverse regions are needed to validate and refine the nomogram for broader populations. Second, key variables, including depressive symptoms, eHealth literacy, and subjective cognitive decline, were assessed using self-reported scales, which may be subject to recall and social desirability biases. Third, the assessment of oral frailty lacked objective clinical dental indicators (e.g., number of teeth, caries status, and presence of prostheses). While the utilized scale is validated, incorporating such clinical measures would strengthen the diagnostic precision and biological plausibility of the findings. Fourth, as previously noted, the use of a convenience sampling method limits the generalizability of our results to the broader older adult population. Future research would benefit from longitudinal or interventional designs, the inclusion of objective clinical oral health assessments, and the adoption of random sampling strategies to verify causal pathways and enhance the external validity of the findings. Furthermore, the model requires validation in independent, prospective cohorts before its clinical utility can be established.

### Conclusion

Our results advocate for a paradigm that views oral frailty as a key sentinel and amplifier of geriatric decline. Effective intervention requires moving beyond a siloed dental approach. Clinical practice and public health strategies should integrate oral health screening into standard geriatric and frailty assessments. Interventions must be equally integrated, combining nutritional support, cognitive and mental health care, social connectivity initiatives, and targeted patient empowerment through health literacy. Breaking the cycle between oral frailty and malnutrition, while supporting psychological wellbeing, represents a critical strategy for preserving overall function and quality of life in aging populations.

## Data Availability

The raw data supporting the conclusions of this article will be made available by the authors, without undue reservation.
